# The T-pod is as stable as supraacetabular fixation using 1 or 2 Schanz screws in partially unstable pelvic fractures: a biomechanical study

**DOI:** 10.1186/s40001-020-00427-0

**Published:** 2020-07-18

**Authors:** Christian Zeckey, Adrian Cavalcanti Kußmaul, Eduardo M. Suero, Christian Kammerlander, Axel Greiner, Matthias Woiczinski, Christian Braun, Wilhelm Flatz, Wolfgang Boecker, Christopher A. Becker

**Affiliations:** 1 Department of General, Trauma and Reconstructive Surgery, University Hospital, Ludwig-Maximilians-University Munich, Marchioninistr. 15, 81377 Munich, Germany; 2 Department of Orthopedic Surgery, Physical medicine and Rehabilitation, University Hospital, Ludwig-Maximilians-University Munich, Munich, Germany; 3 Institute of Legal and Forensic Medicine, University Hospital, Ludwig-Maximilians-University Munich, Munich, Germany; 4 Institute for Radiology, University Hospital, Ludwig-Maximilians-University Munich, Munich, Germany

**Keywords:** Pelvic fracture, Biomechanic, Pelvic bandage, Fracture fixation, External fixator

## Abstract

**Introduction:**

Unstable fractures of the pelvis remain the predominant cause of severe hemorrhage, shock and early death in severely injured patients. The use of pelvic binders has become increasingly popular, particularly in the preclinical setting. There is currently insufficient evidence available about the stability of the pelvic binder versus supraacetabular fixation using 1 or 2 Schanz screws. We aimed to analyze the stability of the pelvic binder and supraacetabular fixateurs using either 1 or 2 Schanz screws in a cadaver model of an induced pelvic B-type fracture.

**Materials and methods:**

The study was undertaken in 7 human fresh-frozen cadaveric pelvises with induced AO-type B fractures. Three stabilization techniques were compared: T-POD (pelvic bandage), supraacetabular external fixator with 1 pin on each side and external fixator with 2 pins on each side. Stability and stiffness were analyzed in a biomechanical testing machine using a 5-step protocol with static and dynamic loading, dislocation data were retrieved by ultrasound sensors at the fracture sites.

**Results:**

No significant differences in fracture fragment displacement were detected when using either the T-POD, a 1-pin external fixator or a 2-pin external fixator (*P* > 0.05). The average difference in displacement between the three methods was < 1 mm.

**Conclusions:**

Pelvic binders are suitable for reduction of pelvic B-type fractures. They provide stability comparable to that of supraacetabular fixators, independently of whether 1 or 2 Schanz screws per side are used. Pelvic binders provide sufficient biomechanical stability for transferring patients without the need to first replace them with surgically applied external fixators. However, soft tissue irritation has to be taken into consideration and prolonged wear should be avoided.

**Level of evidence:**

Level III

## Introduction

Unstable fractures of the pelvis remain the predominant cause of severe hemorrhage, shock and early death in severely injured patients. Emergency fixation of unstable fracture patterns of the pelvis should fulfill various criteria such as reduction of intrapelvic volume and stable preliminary reduction in order to control the associated bleeding and hemorrhagic shock [1].

In recent times, the use of pelvic binders has become increasingly popular, particularly in the preclinical setting. There is evidence that pelvic binders lead to sufficient preliminary reduction and, therefore, control of the bleeding [2]. There is currently insufficient evidence regarding the timing of removal the pelvic binder and whether or not the stability is sufficient for longer timeframes. There are reports of pelvic binders having been left in place after hospital admission and until a skilled pelvic surgeon was available without any complications. Other studies report deleterious effects on the soft tissues, including necrosis with later soft tissue coverage, moreover the assessment of the pelvis in terms of complex pelvic trauma is limited as is the nursing management on ICU [3, 4].

Pelvic binders are routinely removed during the early diagnostic stages after the patient has reached the hospital in favor of external fixator. These are typically mounted in a supraacetabular technique using either 1 or 2 Schanz screws per side [5]. Although some authors argue that 2 Schanz screws provide enhanced stability of the construct, particularly in terms of rotational stability of the ilium, the technique also entails additional damage to the soft tissues and increased time to complete the procedure. Moreover, there is no available data supporting the alleged biomechanical superiority of the 2-pin construct [6].

Although there is consent about the usefulness of pelvic binders in the preclinical and clinical setting, biomechanical investigations are widely missing. In addition, there are no studies directly comparing pelvic binders to supraacetabular fixators using either 1 or 2 Schanz screws in a human cadaver model.

In this study, we aimed to compare the stability of the pelvic binder and supraacetabular fixators using either 1 or 2 Schanz screws in a cadaver model of an induced pelvic B-type fracture.

We hypothesized that 1. the pelvic binder is as stable as the external supraacetabular fixator and 2. that there is no difference when using 1 or 2 Schanz screws per side.

## Materials and methods

This study was conducted with the approval of the ethics committee of the Ludwig Maximilian University of Munich (No. 18-071 UE). A total of 7 human fresh-frozen whole cadaveric pelvises were used. The pelvises were harvested by the Institute of Forensic Medicine of the Ludwig-Maximilians-University of Munich between January 2016 and April 2017. Retrospective approval for donation was given by the donors’ relatives. Exclusion criteria for selection were any existing trauma and consecutive damage to the musculoskeletal system of the donor, a preexisting tumor or tuberculosis disease.

The frozen pelvises were taken out of the freezer (− 20 °C) 1 day before their experiment to ensure steady warm-up to room temperature. 30 min prior to its testing, each pelvis was placed in a hot water bath (approx. 35 °C) to imitate body temperature.

In order to perform the osteotomy of the sacrum and the pubic rami and to install the ultrasound sensors needed for measurement, the soft tissue and muscle were dissected, trying to keep capsules and ligaments intact. The dissection was kept at a minimum including only the spots required, in order to maintain the biomechanical properties of the pelvises.

Prior to the experiment, the bone mineral density (BMD) of all pelvises was measured with quantitative computed tomography (qCT), taking into consideration their fourth and fifth lumbar vertebrae (Table 1).

**Table 1:** Characteristics of the pelvises used in this study

Pelvis	Age (years)	Sex	BMD (mg Ca-Ha/ml)
1	72	Male	113.7
2	25	Male	151.7
3	51	Female	171.0
4	67	Male	63.2
5	60	Male	121.6
6	65	Male	133.7
7	65	Male	64.7
Mean:	57.9 + / − 15.9		117.1 + / − 40.9
Median:	65		121.6

*BMD* bone mineral density

After the preparation, an AO-type B2.2 fracture was created using a thin hacksaw blade, combining a posterior sacral fracture with a fracture of both the superior and the inferior pubic ramus. Next, the different osteosynthesis methods were applied with the pelvises being placed into the test instrument each time and data being recorded for each method individually.

**Fig. 1:**
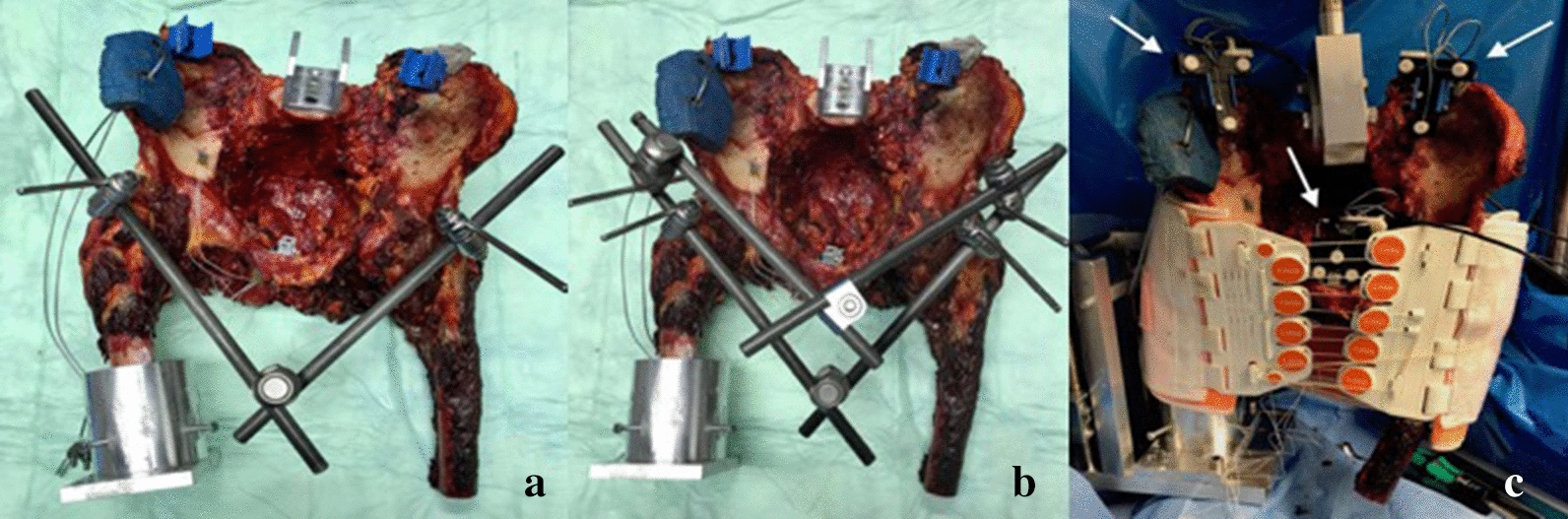
**a**–**c**: Experimental setup. Supraacetabular external fixator with one pin (**a**) and two pins (**b**) on each side. T-POD applied on the cadaveric pelvis (**c**) with arrows indicating the sensors of the 3D-ultrasound measuring system

For this study, the following osteosynthesis methods were examined: T-POD (pelvic binder), supraacetabular external fixator with 1 pin on each side, and external fixator with 2 pins on each side (Fig. 1a–c). All tests were performed by two trauma specialists at all times.

Motion data were recorded using a 3D ultrasound measuring system (Zebris CMS20, Gilching, Germany). The system consisted of 3 sensors placed onto the pelvis as shown in Fig. 1c. Two sensors are mounted bilateral on the iliac crest about 5 cm lateral to the superior posterior iliac spine and one sensor was mounted next to the pubic symphysis. The transducer was positioned 50 cm anterior to the pelvis. Between the 3 sensors the change of the relative distance was measured during biomechanical loading to analyze anterior and posterior displacement.

The pelvises were inserted into an all-electric testing machine (Instron ElectroPulsTM E10000 Linear-Torsion, Norwood, MA 02062-2643, USA). The femora of the fractured side of the pelvises were embedded into a metal cylinder containing epoxide resin in order to simulate a single leg stance.

To ensure comparability of all trials, a 5-step protocol was applied to all pelvises (Table 2) modified from McDonald, Becker and Suero et al. [7–10]. The position of the ultrasound sensors was recorded every 30 ms (resolution 0.1 mm), enabling the calculation of the relative distance between all sensors at all times. The relative distances between all sensors were then also taken into consideration for the final analysis.

**Table 2:** Testing protocol for comparing pelvic binder and external fixation techniques for pelvic injuries

Step 1	Loading up to 150 N
Step 2	Holding at 150 N for 30 s
Step 3	Periodic loading: 20 cycles with a frequency of 0,25 Hz between 150N and 250N
Step 4	Holding at 150 N for 30 s
Step 5	System back to its original position of +28 mm

Displacement of the posterior and anterior fracture site was measured (mm) and stiffness was calculated as mean force divided through mean displacement (N/mm).

## Statistical analysis

We used linear regression to model the magnitude of displacement at the posterior fracture (ileum–sacrum/ileum–ileum) and the anterior fracture (ileum–symphysis) as a function of the three stabilizing techniques used (1-pin fixation, 2-pin fixation and T-POD). Clustered standard errors were calculated using the Huber–White method. Pairwise comparisons were carried out using *t*-tests. The Holm method was used to adjust the *P*-values for multiple comparisons. For all tests, α was set to 0.05. Descriptive statistics are presented as mean ± standard deviation (SD) wherever appropriate.

## Results

The characteristics of the pelvises used in this experiment are shown in Table 1. Mean BMD was 117.1 ± 40.9 mg Ca-Ha/ml and mean age of the pelvis donors was 58 ± 16 years.

No significant differences in fracture fragment displacement were detected when using either the T-POD (posterior: 2.3 ± 0.8 mm, anterior: 2.5 ± 1.1 mm), a 1-pin external fixator (posterior 1.9 ± 0.6 mm, anterior: 2.6 ± 1.4 mm) or a 2-pin external fixator (posterior: 2.2 ± 0.7 mm, anterior: 2.4 ± 0.9 mm) (*P* > 0.05). Also no significant differences showed the mean stiffness (*P* > 0.05): T-POD (posterior: 49.3 ± 16.4 N/mm, anterior: 47.9 ± 18.2 N/mm), 1-pin external fixator (posterior 57.0 ± 17.9 N/mm, anterior: 47.3 ± 21.3 N/mm), 2-pin external fixator (posterior: 50.8 ± 14.1 N/mm, anterior: 46.7 ± 17.2 N/mm).

**Fig. 2:**
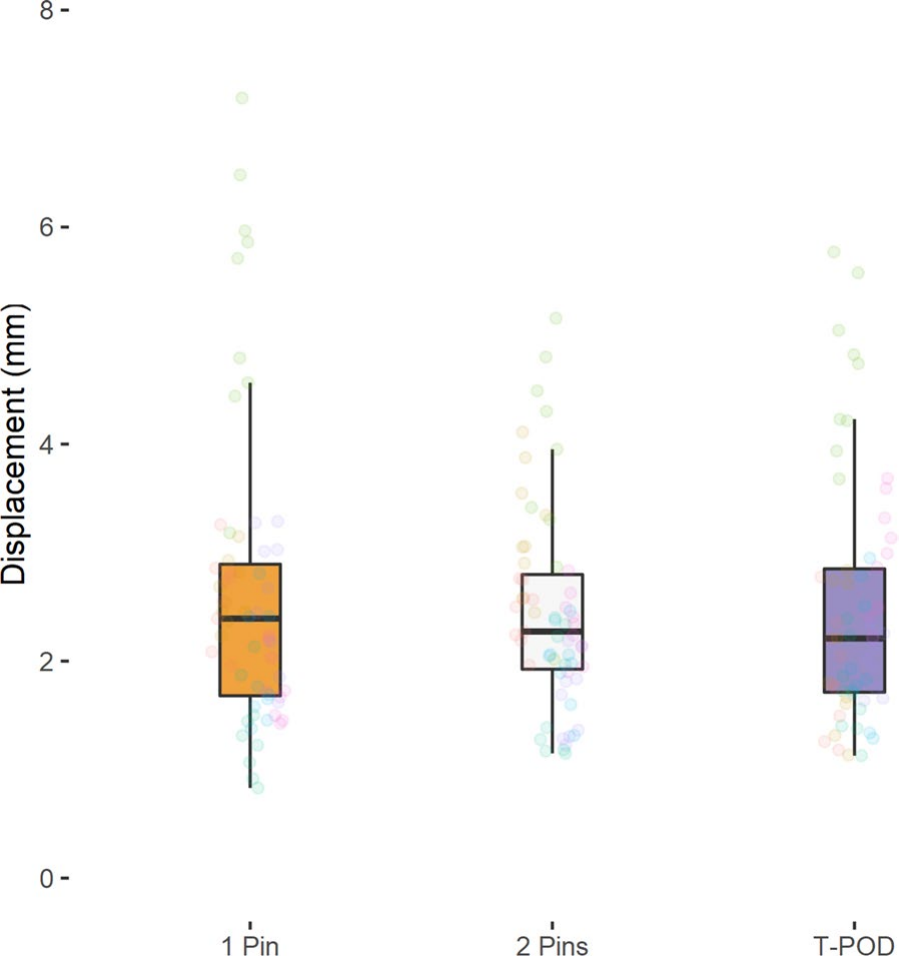
Anterior fracture displacement ileum–symphysis with each fixation technique

**Fig. 3:**
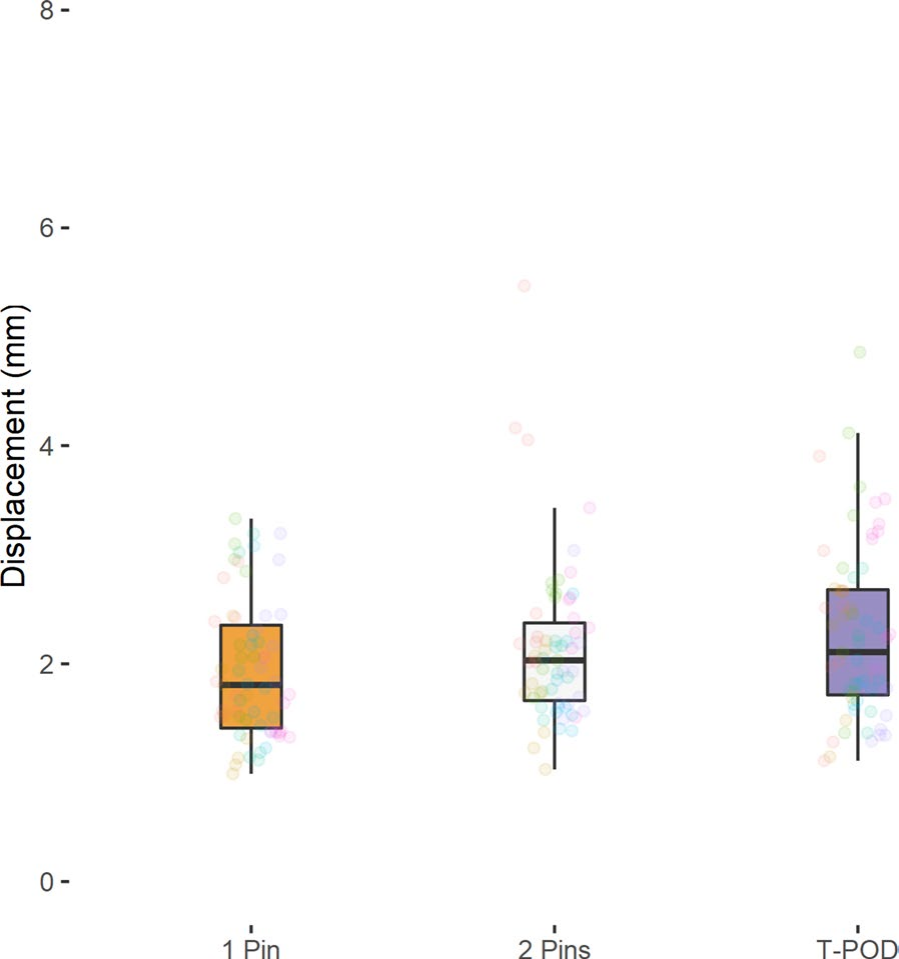
Posterior fracture displacement ileum–sacrum (ileum–ileum) with each fixation technique

**Fig. 4:**
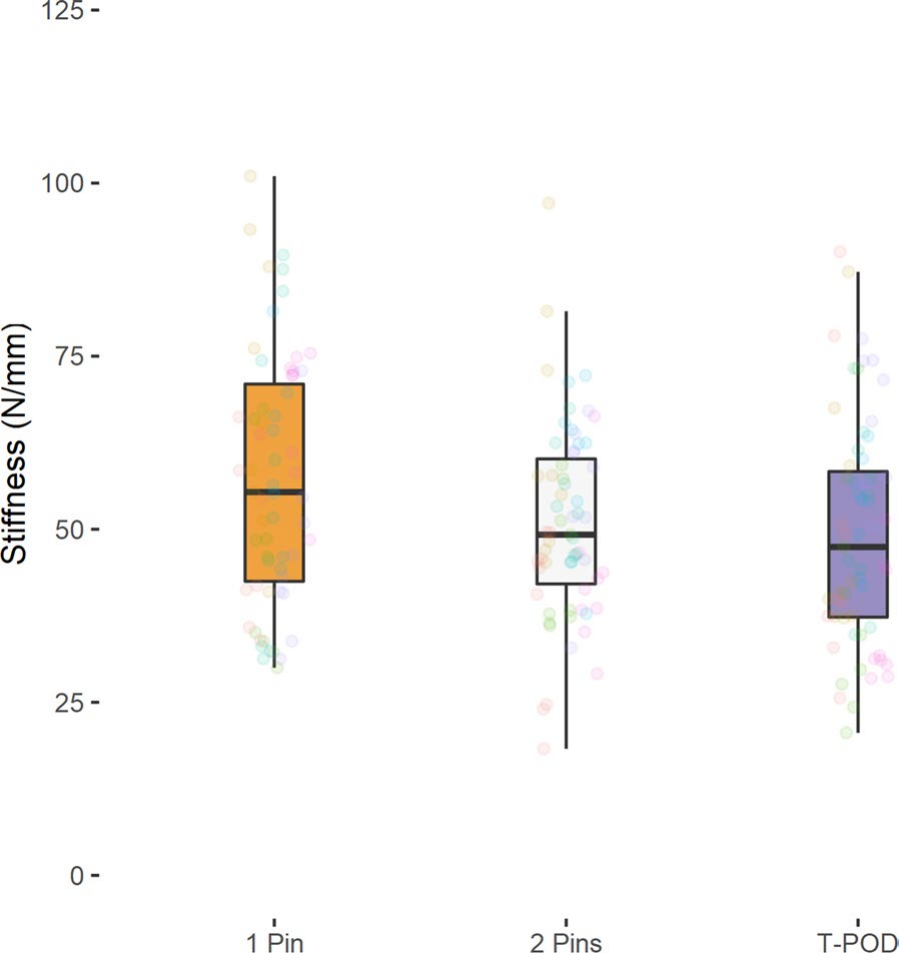
Posterior stiffness ileum–sacrum (ileum–ileum) with each fixation technique

**Fig. 5:**
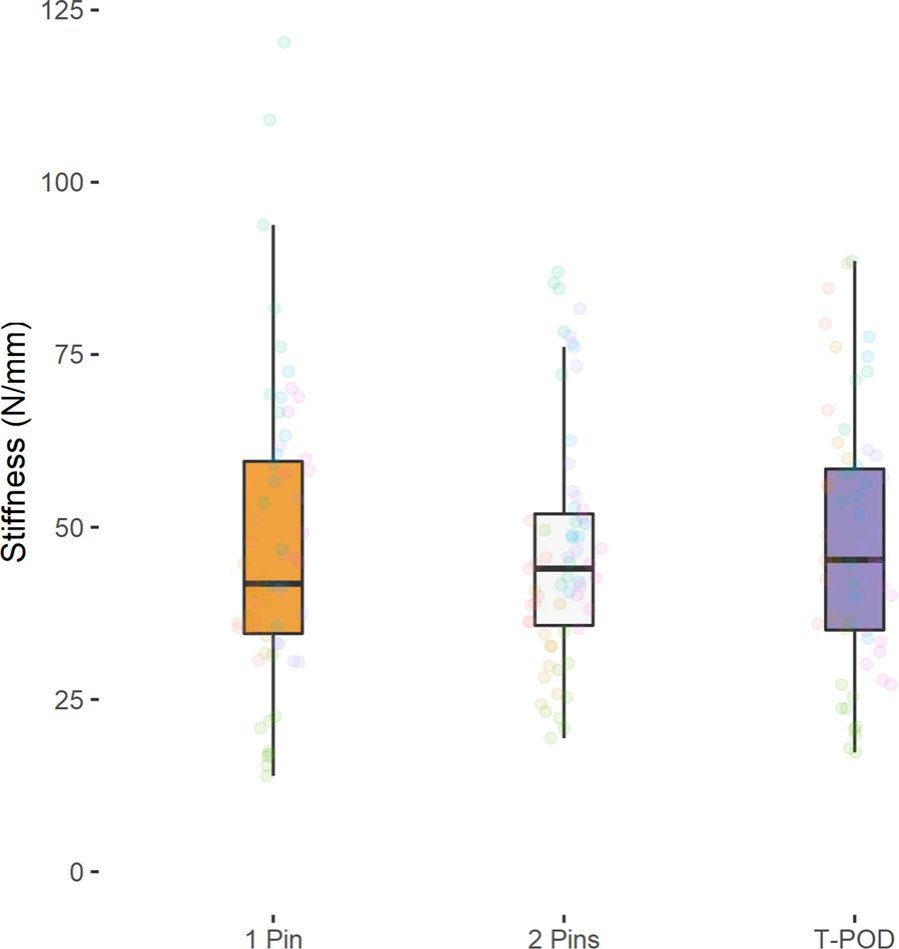
Anterior stiffness ileum–symphysis with each fixation technique

Tables 3, 4, 5 as well as Figs. 2, 3, 4, 5 summarize the mean displacement and stiffness at the ileum–symphysis (anterior fracture) and the ileum–sacrum (ileum–ileum) (posterior fracture) with each fixation technique.

**Table 3:** Mean displacement and stiffness at the fracture site when using the 1-pin and 2-pin external fixator techniques and the T-POD pelvic binder for stabilization of pelvic injuries

	1-pin	2-pin	T-POD
Mean displacement (mm)			
Posterior	1.94 ± 0.64	2.15 ± 0.73	2.27 ± 0.80
Anterior	2.60 ± 1.35	2.44 ± 0.91	2.46 ± 1.11
Mean stiffness (N/mm)			
Posterior	57.04 ± 17.82	50.76 ± 14.07	49.26 ± 16.36
Anterior	47.34 ± 21.30	46.66 ± 17.17	47.89 ± 18.16

**Table 4:** Pairwise comparison of mean displacement between the different pelvic fracture fixation techniques

Region	Comparison	Mean displacement difference (mm)	Std. error	95% CI	*P*-value	Adj. *P*-value
Ileum–sacrum (Ileum-Ileum)	2 pins vs. 1 pin	− 0.21	0.18	− 0.57, 0.15	0.2521	0.7563
	T-POD vs. 1 pin	0.33	0.23	− 0.12, 0.78	0.1444	0.4332
	T-POD vs. 2 pins	0.13	0.16	− 0.19, 0.44	0.4394	1
Ileum–symphysis	2 pins vs. 1 pin	0.16	0.28	− -0.39, 0.72	0.5616	1
	T-POD vs. 1 pin	− 0.14	0.25	− 0.64, 0.36	0.5764	1
	T-POD vs. 2 pins	0.02	0.25	− 0.47, 0.51	0.9327	1

**Table 5:** Pairwise comparison of mean stiffness between the different pelvic fracture fixation techniques

Region	Comparison	Mean displacement difference (mm)	Std. error	95% CI	*P*-value	Adj. *P*-value
Ileum–sacrum (Ileum-Ileum)	2 pins vs. 1 pin	6.27	3.89	− 1.41, 13.95	0.1089	0.3267
	T-POD vs. 1 Pin	− 7.78	5.52	− 18.67, 3.12	0.1609	0.4827
	T-POD vs. 2 pins	− 1.5	3.14	− 7.69, 4.68	0.6321	1
Ileum–symphysis	2 pins vs. 1 pin	0.68	4.63	− 8.45, 9.81	0.8836	1
	t-pod vs. 1 pin	0.54	5.69	− 10.68, 11.77	0.9239	1
	T-POD vs. 2 pins	1.22	4.65	− 7.96, 10.41	0.793	1

Similar mean displacement and stiffness values were recorded after axial loading the pelvic fracture models stabilized using the 1-pin, 2-pin and T-POD pelvic injury stabilization techniques.

Pairwise testing showed non-significant differences of less than 1 mm in mean displacement between the three techniques at both the anterior and posterior pelvic landmarks (*P* > 0.05) (Table 4).

No significant differences in mean stiffness were detected between the techniques (*P* > 0.05).

## Discussion

In the current study, we aimed to investigate the stability of the pelvic binder compared to supraacetabular fixators using either 1 or 2 Schanz screws per side in B-type fractures of the pelvis in a cadaver model. The main findings of the study were that anterior displacement of the pelvis was comparable when using any of the three fixation methods and that no difference in the posterior stability of the pelvic ring was detected between the three methods.

- No differences were found between using 1 or 2 Schanz screws per side when fixing a B-type fracture of the pelvis using a supraacetabular external fixator.

Unstable fractures of the pelvis are challenging injuries due to their high risk of bleeding and associated risk of death. Reduction and retention in later stages is a technically highly demanding procedure. Immediate stabilization even in the preclinical setting is necessary in order to reduce the intrapelvic volume which might lead to control of the hemodynamic situation. In recent times, pelvic binders have become increasingly popular in the preclinical and emergency medical services (EMS) settings. In some areas, pelvic binders are applied based on the mechanism of injury and patient condition independent of the clinical instability of the pelvis [11, 12]. The timing of removal of the pelvic binder once the patient has reached the hospital is an ongoing topic of debate. In certain circumstances, the pelvic binder leads to limited visibility of the fracture during the first diagnostic steps, particularly in open book-type injuries. Other bony injuries, such as pelvic C-type fractures, are usually visible even with the pelvic binder in place.

One has to re-check the correct application of the binder since necessary manipulations of the patient in ICU wards might lead to dislocation of the binder and to insufficient stability. This aspect has been evaluated in a study by Prasarn et al. The investigators examined the position of the T-POD either on the level of the greater trochanter or on the iliac spine in a cadaver model. They were able to demonstrate that there was less motion in their experimental setup when the T-POD was applied at the level of the greater trochanter [13]. Another study investigated the Sam Sling at three levels of application. The investigators were able to demonstrate that there was less tension required when the device was positioned around the greater trochanters [14].

There is an ongoing debate about the timeframe of removal of the pelvic binder in favor of surgically applied stabilization devices, such as external fixators. There are reports about binders that were kept in place for several hours to days without any complications; however, severe complications such as soft tissue necrosis and recurrent hemodynamic instability have also been reported [2, 15, 16]. In an experimental setup, skin pressure patterns by pelvic binder devices were investigated when reducing a rotationally unstable fracture in a cadaver setup. Maximum pressure was 255–308 mmHg while mean pressures were as high as 173–233 mmHg, which reflects the potentially harmful effect of the device on the soft tissues [12]. Given these results, an unnecessarily prolonged wear of the devices should be avoided.

In addition, the stability of the construct compared to supraacetabular external fixators has remained an open question. To the best of our knowledge, there are very few studies available investigating the stability patterns when using external fixation and pelvic binders. In the available studies, external fixation was obtained through the use of iliac crest external fixators, which are known to provide less stability compared to supraacetabular positioning. In a study by Prasarn et al., it was shown that the T-POD was able to provide comparable results to those of external fixation of the ilium [17]. Our results confirm the stability of the pelvic binder compared to the supraacetabular external fixator, independently of whether 1 or 2 Schanz screws per side are used.

Supraacetabular fixators are frequently used in unstable pelvic fractures due to their fast application and effective preliminary or sometimes definitive stabilization. Some authors argue that there is a need for using 2 Schanz screws per side, whereas others believe that 1 screw per side is sufficient. The argument in favor of using 2 screws per side is increased stability of the fracture. However, the procedure takes more time and an increased affection of the soft tissues, including the lateral femoral cutaneous nerve, is evident.

In our study we were able to demonstrate that the pelvic binder produces comparable stability as it is provided by supraacetabular external fixators.

To the best of our knowledge, this is the first study describing the biomechanical context of pelvic external fixation, which might affect clinical pathways. Since we know that the pelvic binder produces sufficient stability that is comparable to supraacetabular external fixators with 1 or 2 screws per side, it seems reasonable to keep the binder in place whenever there is no hemodynamic instability and there is a need for further interventions such as craniotomies or thoracotomies. It may also be an option to keep the binder in the correct place in case of transferal of the patients from rural areas to higher level trauma centers. Based on the results of the current study, there is no need to first fix the pelvis surgically prior to transferring a patient. Since it is known that secondary transfer of multiple trauma patients to level-1 trauma centers is frequently based on specific injuries, such as those of the pelvis, the correctly applied pelvic binder might serve as an excellent option for temporary stabilization in transfer patients [18]. Whenever there is a pelvic binder in place in pelvic fractures, the position of the binder has to be re-evaluated closely. An incorrect application will lead to re-dislocation of the fracture and potentially recurrent hemodynamic instability. The position of the pelvic binder has to be re-assessed frequently since the position is crucial in maintaining proper reduction forces [13].

In cases of hemodynamic instability due to the pelvic fracture, retroperitoneal packing or selective angioembolization is indicated after achieving stable osseous reduction [19]. Since there is no surgical access to the retroperitoneum with the binder in place, external fixation and removal of the binder is necessary in those cases. Also nursing of multiple trauma patients on the ICU ward is limited due to both the potential for dislocation of the binder and access to the soft tissues including the urogenital area. Another potential disadvantage is the limited access to the soft tissues and, therefore, limited clinical examination. This is particularly relevant in complex fractures of the pelvis with associated injuries.

The study at hand provides both, strengths and limitations. The strengths of the study are the reproducible fracture patterns and the standardized measurement of the dislocation. Moreover, this study has been performed in human fresh-frozen pelvises, which might represent a closer and more realistic scenario than artificial bone models. However, the relatively small sample size is a drawback that is based on the limited availability of fresh-frozen human pelvises and is comparable to that of previous studies. A further limitation is that the study was carried out in a model without soft tissues and fractures were induced by sawblades, which is not the case in the clinical setting. However, this method ensures the reproducibility of the fracture patterns across specimens, which is paramount for precisely addressing the hypotheses of this study. A final limitation is that we cannot answer the question about differences in stability when using the investigated devices in other randomly induced fracture line types. However, due to our standardized setting, accurate and technically sound measurements and the study setup, we feel safe in drawing conclusions out of the data of our study.

## Conclusions

Pelvic binders are suitable for reduction of pelvic B-type fractures. They provide stability comparable to that of supraacetabular fixators, independently of whether 1 or 2 Schanz screws per side are used. Pelvic binders provide sufficient biomechanical stability for transferring patients without the need to first replace them with surgically applied external fixators. However, soft tissue irritation has to be taken into consideration and prolonged wear should be avoided.

## Data Availability

The datasets used and/or analyzed during the current study are available from the corresponding author on reasonable request.
